# Draft Genome Sequence of *Streptomyces* sp. TP-A0874, a Catechoserine Producer

**DOI:** 10.1128/genomeA.01163-16

**Published:** 2016-10-20

**Authors:** Hisayuki Komaki, Akira Hosoyama, Natsuko Ichikawa, Yasuhiro Igarashi

**Affiliations:** a Biological Resource Center, National Institute of Technology and Evaluation (NBRC), Chiba, Japan; b NBRC, Tokyo, Japan; c Biotechnology Research Center and Department of Biotechnology, Toyama Prefectural University, Toyama, Japan

## Abstract

We report the draft genome sequence of *Streptomyces* sp. TP-A0874 isolated from compost. This strain produces catechoserine, a new catecholate-type inhibitor of tumor cell invasion. The genome harbors at least six gene clusters for polyketide and nonribosomal peptide biosyntheses. The biosynthetic gene cluster for catechoserines was identified by bioinformatic analysis.

## GENOME ANNOUNCEMENT

In our search for novel bioactive compounds from actinomycetes, a phylogenetically novel strain, *Streptomyces* sp. TP-A0874, was isolated from a compost sample collected in Ishikawa, Japan, and found to produce a new catecholate derivative designated catechoserine, along with two known biosynthetically related metabolites, *N*,*N*′-bis(2,3-dihydroxybenzoyl)-*O*-l-seryl-l-serine and *N*,*N*′,*N*′-tris(2,3-dihydroxybenzoyl)-*O*-l-seryl-*O*-l-seryl-l-serine. These three compounds share an *N*-2,3-dihydroxybenzoyl-L-serine as a common structural unit and inhibit the invasion of murine colon carcinoma 26-L5 cells (1). To identify the biosynthetic gene cluster for these compounds and assess the potential to produce other secondary metabolites, we sequenced the genome of *Streptomyces* sp. TP-A0874 and surveyed biosynthetic gene clusters for nonribosomal peptides and polyketides.

*Streptomyces* sp. TP-A0874 was deposited into the NBRC culture collection and is publicly available as NBRC 110466. The whole genome of *Streptomyces* sp. TP-A0874 monoisolate was read by using a combined strategy of shotgun sequencing with GS FLX+ (Roche; 84 Mb of sequences, 16-fold coverage) and paired-end sequencing with MiSeq (Illumina; 794 Mb of sequences, 153-fold coverage). These reads were assembled using Newbler version 2.8 and subsequently finished using GenoFinisher (2), which led to a final assembly of 16 scaffold and two contig sequences of >500 bp each. The total size of the assembly was 5,097,807 bp, with a G+C content of 71.4%. Coding sequences were predicted by Prodigal (3). Nonribosomal peptide synthetase (NRPS) and polyketide synthase (PKS) gene clusters were analyzed in the same manner previously reported (4).

The genome contains at least four NRPS gene clusters, one type II PKS gene cluster, and one hybrid PKS/NRPS gene cluster. One of three NRPS gene clusters in scaffold00002 was annotated to be responsible for the synthesis of catechoserine and its related metabolites, because the domain organizations of orf342, orf340, and orf339 in the cluster are adenylation for dihydroxybenzoate, thiolation, and condensation-adenylation for serine-thiolation-thioesterase, respectively, which is in accordance with the chemical structure of catechoserines. The other two NRPS gene clusters in scaffold00002 are predicted to synthesize a siderophore comprising serine and ornithine and a dipeptide including alanine, respectively. A large NRPS gene cluster is present in the genome, but it was not completely sequenced and was divided into scaffold00010 and scaffold00014. The type II PKS gene cluster in scaffold00004 may synthesize tetracenomycin-like compounds, because its ketosynthase alpha (KSα) and KSβ (chain length factor) showed 85% and 74% sequence identities to those annotated to tetracenomycin C polyketide beta-ketoacyl synthases in *Streptomyces davawensis* JCM 4913. The hybrid PKS/NRPS gene cluster in scaffold000001 may synthesize compound(s) derived from a C_10_-polyketide chain and a threonine molecule, because it has five PKS modules and an NRPS module incorporating threonine.

Bioactive compounds other than catechoserines have not been isolated from the strain. However, the presence of NRPS and PKS gene clusters suggests that this strain can produce nonribosomal peptides and polyketides. The information reported here will help further the discovery of uncharacterized secondary metabolites.

### Accession number(s).

The draft genome sequence of *Streptomyces* sp. TP-A0874 has been deposited in the DDBJ/ENA/GenBank database under the accession no. BBZK00000000. The version described in this paper is the first version, BBZK01000000.
